# Ancient Geographical Barriers Drive Differentiation among *Sonneratia caseolaris* Populations and Recent Divergence from *S. lanceolata*

**DOI:** 10.3389/fpls.2016.01618

**Published:** 2016-10-26

**Authors:** Yuchen Yang, Norman C. Duke, Fangfang Peng, Jianfang Li, Shuhuan Yang, Cairong Zhong, Renchao Zhou, Suhua Shi

**Affiliations:** ^1^State Key Laboratory of Biocontrol and Guangdong Provincial Key Laboratory of Plant Resources, Sun Yat-Sen UniversityGuangzhou, China; ^2^Trop WATER, James Cook University, TownsvilleQLD, Australia; ^3^Hainan Dongzhai Harbor National Nature ReserveHaikou, China

**Keywords:** genetic differentiation, secondary contact, Pleistocene glaciations, *Sonneratia*, mangroves

## Abstract

Glacial vicariance is thought to influence population dynamics and speciation of many marine organisms. Mangroves, a plant group inhabiting intertidal zones, were also profoundly influenced by Pleistocene glaciations. In this study, we investigated phylogeographic patterns of a widespread mangrove species *Sonneratia caseolaris* and a narrowly distributed, closely related species *S. lanceolata* to infer their divergence histories and related it to historical geological events. We sequenced two chloroplast fragments and five nuclear genes for one population of *S. lanceolata* and 12 populations of *S. caseolaris* across the Indo-West Pacific (IWP) region to evaluate genetic differentiation and divergence time among them. Phylogenetic analysis based on sequences of nuclear ribosomal internal transcribed spacer and a nuclear gene *rpl9* for all *Sonneratia* species indicate that *S. lanceolata* individuals are nested within *S. caseolaris*. We found strong genetic structure among geographic regions (South China Sea, the Indian Ocean, and eastern Australia) inhabited by *S. caseolaris*. We estimated that divergence between the Indo-Malesia and Australasia populations occurred 4.035 million years ago (MYA), prior to the onset of Pleistocene. BARRIERS analysis suggested that complex geographic features in the IWP region had largely shaped the phylogeographic patterns of *S. caseolaris*. Furthermore, haplotype analyses provided convincing evidence for secondary contact of the South China Sea and the Indian Ocean lineages at the Indo-Pacific boundary. Demographic history inference under isolation and migration (IM) model detected substantial gene flow from the Sri Lanka populations to the populations in the Java Island. Moreover, multi-locus sequence analysis indicated that *S. lanceolata* was most closely related to the Indian Ocean populations of *S. caseolaris* and the divergence time between them was 2.057 MYA, coinciding with the onset of the Pleistocene glaciation. Our results suggest that geographic isolation driven by the Pleistocene ice age resulted in the recent origin of *S. lanceolata*.

## Introduction

Pleistocene glaciations have profoundly shaped the biogeography of extant global flora and fauna (Hewitt, 2000, 2001). While tropical animals and plants were less influenced than their temperate counterparts by temperature fluctuations during this period, dramatic sea level changes that accompanied glaciation affected organisms inhabiting tropical marine or intertidal zone (Barber et al., 2000; Kittiwattanawong et al., 2001; Timm and Kochzius, 2008; Macieira et al., 2015). Indo-Australian Archipelago (IAA), which extends from the Malay Peninsula through Sumatra, Borneo and Java to Sulawesi and the island of New Guinea, has the richest biodiversity on the planet (Mora et al., 2003). Molecular phylogenetic and population genetic studies in many marine organisms have revealed strong genetic discontinuities between the bodies of water separated by IAA (McMillan and Palumbi, 1995; Williams and Benzie, 1998; Duda and Palumbi, 1999; Nelson et al., 2000; Kochzius et al., 2003; Froukh and Kochzius, 2008). Barber et al. (2000) proposed a marine Wallace’s line according to the genetic breaks between populations from north and south of Flores and Java Seas, and suggested that Pleistocene glacial isolation acted as a driver of intra-species genetic differentiation.

Mangroves are the dominant woody plants in the intertidal zones of the tropics (Tomlinson, 1986). Like other organisms inhabiting tropical marine or intertidal zones, they exhibit a strong association of genetic divergence with geographical breaks (Duke, 1995; Dodd and Rafii, 2002; Triest, 2008). Mangrove species are split into two main biogeographic regions of species diversity, the Atlantic-East Pacific (AEP) and the Indo-West Pacific (IWP; Tomlinson, 1986; Duke et al., 1998). Within the IWP region, there are two biodiversity centers, Indo-Malesia (Indian subcontinent and Southeast Asia) and Australasia (from Australia and New Guinea to west Polynesia) (Ricklefs and Latham, 1993), which may be evolutionarily distinct (Daru et al., 2013). Although the majority of species are shared between the two regions (Hogarth, 1999, 2001), there are 7–9 species exclusively distributed in Australasia, while 11 are exclusive to Indo-Malesia (Duke et al., 1998). Genetic breaks between populations from the two regions have been observed in many mangrove species, such as *Lumnitzera littorea* (Su et al., 2007), *Bruguiera gymnorrhiza* (Urashi et al., 2013), and *Rhizophora mucronata* (Wee et al., 2015). Understanding the linkage between intra- and inter-species genetic diversity on one hand, and historical geological events on the other hand should provide important insights into the evolutionary mechanisms of biodiversity generation in mangrove plants.

*Sonneratia*, a typical mangrove genus widely distributed across the IWP, comprises six species and three hybrid taxa (Duke, 1984, 1994; Wang and Chen, 2002; Zhou et al., 2005; Qiu et al., 2008). *Sonneratia caseolaris* is a wide-spread species, ranging from Sri Lanka through the Malay Peninsula to China and Australia (Tomlinson, 1986; Duke, 2006). It grows in upstream reaches of river-dominated estuaries with low levels of salinity and a high supply of fresh water (Duke and Jackes, 1987; Duke et al., 1998). Its sibling species, *S. lanceolata*, has a high similarity in morphology and ecological requirement with *S. caseolaris* (Duke, 2006). Both species have red petals, flat and expanded calyx lobes, distinct mucronate leaf apex and usually occur in the upstream of rivers (Duke, 1994, 2006). However, their geographical distributions do not overlap (Duke and Jackes, 1987). *S. lanceolata* is restricted to northwestern Australia, southern New Guinea and few locations in Indonesia. In contrast, *S. caseolaris* is found in the Indo-Malesia region, northeastern Australia and northern New Guinea (Duke, 2006). These two species thus provide an ideal system to study the impact of Pleistocene sea-level changes on speciation of mangrove plants. However, previous intrageneric classifications of *Sonneratia* based on either morphological characteristics (Ko, 1985, 1993; Wang and Chen, 2002) or molecular data didn’t take *S. lanceolata* into account (Shi et al., 2000; Zhou et al., 2005; Yang et al., 2015), thus, phylogenetic relationship between *S. lanceolata* and *S. caseolaris* remains unclear.

In this study, we first determined the phylogenetic position of *S. lanceolata* in *Sonneratia* using sequences of the nuclear ribosomal internal transcribed spacer (nrITS) and a nuclear gene *rpl9*. We then sequenced two chloroplast fragments and five nuclear genes in samples from one population of *S. lanceolata* and 12 populations of *S. caseolaris* to assess the genetic diversity within *S. caseolaris* and divergence between it and *S. lanceolata*. Our results provide new insights into population dynamics and inter-species divergence of mangrove plants under the influence of sea-level fluctuations during the Pleistocene epoch.

## Materials and Methods

### Plant Materials

We sampled 16 individuals from a single population of *S. lanceolata* in Northern Territory, Australia, and five to 20 individuals from 12 populations of *S. caseolaris* across the IWP region. These 12 populations include four populations around the South China Sea (SCS), two at the Indo-Pacific boundary, two from Java Island, Indonesia, three from the coasts of the Indian Ocean, and one from Queensland, Australia. We collected leaf tissue from each individual and stored it in a plastic bag with silica gel for DNA extraction. The details of sampling locations and sample sizes are presented in **Table 1**.

**Table 1:** Locations and sample sizes of the 12 *Sonneratia caseolaris* populations and the single *S. lanceolata* population used in this study.

Species	Code	Locations	Longitude	Latitude	Sample size
*S. caseolaris*	CWC	Wenchang, Hainan, China	110°46′ E	19°31′ N	19
	CBA	Boao, Hainan, China	110°34′ E	19°9′ N	17
	TST	Surat thani, Thailand	99°19′ E	09°08′ N	21
	MSB	Sibu, Salawa, Malaysia	111°22′ E	2°15′ N	18
	MKK	Kukup, Johor, Malaysia	103°27′ E	1°21′ N	20
	MSN	Merbok, Kedah, Malaysia	100°21′ E	6°07′ N	16
	BPF	Pazra Futo, Sundarban, Bangladesh	89°40′ E	22°12′ N	7
	BSK	Supati Khal, Sundarban, Bangladesh	89°46′ E	21°55′ N	12
	SRK	Rekawa, Sri Lanka	80°51′ E	21°55′ N	20
	ISG	Sungaibuntu, Java, Indonesia	107°25′ E	6°4′ S	19
	ICJ	Cilacap, Java, Indonesia	108°59′ E	7°43′ S	20
	AQS	Daintree River, Australia	101°54′ E	16°16′ S	21
*S. lanceolata*	SLA	South Alligator River, Northern Territory, Australia	132°22′ E	12°23′ S	16

### DNA Extraction, Polymerase Chain Reaction (PCR) Amplification, and Sequencing

Total genomic DNA of each individual was extracted using the CTAB method (Doyle and Doyle, 1987). To resolve the phylogenetic position of *S. lanceolata* within the genus *Sonneratia*, nrITS and one nuclear gene *rpl9* were amplified from two randomly chosen individuals from each population of *S. caseolaris* and *S. lanceolata* by polymerase chain reaction (PCR) with LA Taq DNA polymerase (Takara Bio, Inc., Shiga, Japan). Additionally, two chloroplast fragments (*trn* L – *trn* F and *trn* V – *trn* M) and five nuclear genes (*rpl9, cpi, ppi, phi*, and *nhx2*) were amplified from each individual of *S. lanceolata* and *S. caseolaris* to assess the patterns of genetic diversity between and within the two species. The primers for the nuclear genes were from Zhou et al. (2007). PCR products were purified using 2% agarose gel electrophoresis and extracted using the StarPrep Gel Extraction Kit (GeneStar Biosolutions, Co., Ltd, Beijing, China). The purified products were sequenced using the Sanger method in an ABI 3730 DNA automated sequencer with BigDye Terminator Cycle Sequencing Ready Reaction Kit (Applied Biosystems, Foster city, CA, USA) using the same sets of primers as were used for PCR amplification. All the primers used in this study were presented in **Supplementary Table S1**.

### Phylogenetic Analysis

To identify the phylogenetic position of *S. lanceolata* within the genus, phylogenetic analyses of all six *Sonneratia* species and the outgroup *Duabanga grandiflora* were carried out using the combined sequence data of nrITS and the nuclear gene *rpl9*, as only these two fragments could be successfully amplified in *D. grandiflora*. The partition-homogeneity test suggests that these two fragments can be combined for phylogenetic analysis in the *Sonneratia* genus (*p* = 0.624; Farris et al., 1995). The sequences from four other *Sonneratia* species, *S. alba, S. ovata, S. griffithii*, and *S. apetala*, and the outgroup *D. grandiflora* were downloaded from the NCBI database. GenBank accessions of all the sequences used for phylogenetic tree construction were listed in **Supplementary Table S2**. The sequences of these two fragments were aligned in CLUSTALX (Thompson et al., 1997), followed by manual adjustments in SeqMan v. 7.10 (DNAStar, London, UK). The phylogeny was reconstructed using two methods: maximum parsimony (MP) and maximum likelihood (ML). The analyses were performed in Mega v. 6.06 (Tamura et al., 2013). The MP analysis was carried out using the Tree-Bisection-Recombination (TBR) algorithm (Nei and Kumar, 2000) on random trees with 100 random addition replicates. The Maxtree parameter was set to 500. Statistical support for nodes was evaluated by bootstrap support (BS) values from 1000 replications. *D. grandiflora* was used to root the phylogenetic trees. Consistency index (CI) and retention index (RI) were also employed to estimate homoplasy. Indels were ignored. For ML analysis, TrN + I was selected as the appropriate nucleotide substitution model from a set of 56 models using the Akaike information criterion, as implemented in Modeltest 3.7 (Posada and Buckley, 2004). ML trees were constructed with Nearest-Neighbor-Interchange (NNI) and the confidence measures of nodes were estimated by BS values from 1000 replications.

### Sequence Data Analyses

The sequences of chloroplast fragments and nuclear genes were aligned and edited in SeqMan v. 7.10. The two chloroplast fragments were concatenated for further analyses. Haplotypes of the combined chloroplast fragments and each nuclear gene were phased and validated in DnaSP v. 5.10 (Librado and Rozas, 2009). For each population, the number of segregating sites (S) and haplotypes (H), haplotype diversity (Hd), nucleotide diversity (𝜃_π_) and DNA polymorphism (𝜃_w_, Watterson, 1977) were calculated for the combined chloroplast fragments and each of the five nuclear genes using DnaSP v. 5.10. Haplotype networks were constructed in NETWORK v. 4.6.1.1 (Fluxus Technology, Ltd, Suffolk, UK) using the median-joining algorithm (Bandelt et al., 1999).

To assess genetic divergence between *S. caseolaris* and *S. lanceolata*, as well as among populations of *S. caseolaris*, we performed a Bayesian clustering analysis, implemented in STRUCTURE v. 2.3.3 (Pritchard et al., 2000), using the polymorphic sites of the five nuclear genes from all the populations of these species we sampled. The likelihood for K (ranging from 1 to 8) clusters was computed with 20 replicates per K. Each run had a burn-in of 200,000 iterations, followed by 1,000,000 Markov chain Monte Carlo (MCMC) iterations based on the admixture model and correlated allele frequencies model (Falush et al., 2003). The most likely number of clusters was estimated using the ΔK statistic (Evanno et al., 2005). Graphics were produced using DISTRUCT v.1.1 (Rosenberg, 2004).

To assess among-population sequence divergence, pairwise Kimura two parameter distances and K_XY_ (the average number of nucleotide differences between populations) were calculated at each locus using Mega v. 6.06 and DnaSP v. 5.10, respectively. Multidimensional scaling (MDS) analysis was performed based on the pairwise Kimura two-parameter distances in R v. 3.1.3^1^. For both species, only the individuals without any missing data were included in PCA. Starting from K_XY_ estimates, putative geographic barriers in the IWP region were inferred for all 13 populations using Monmonier’s algorithm in the software Barrier v. 2.2 (Manni et al., 2004).

**FIGURE 1:**
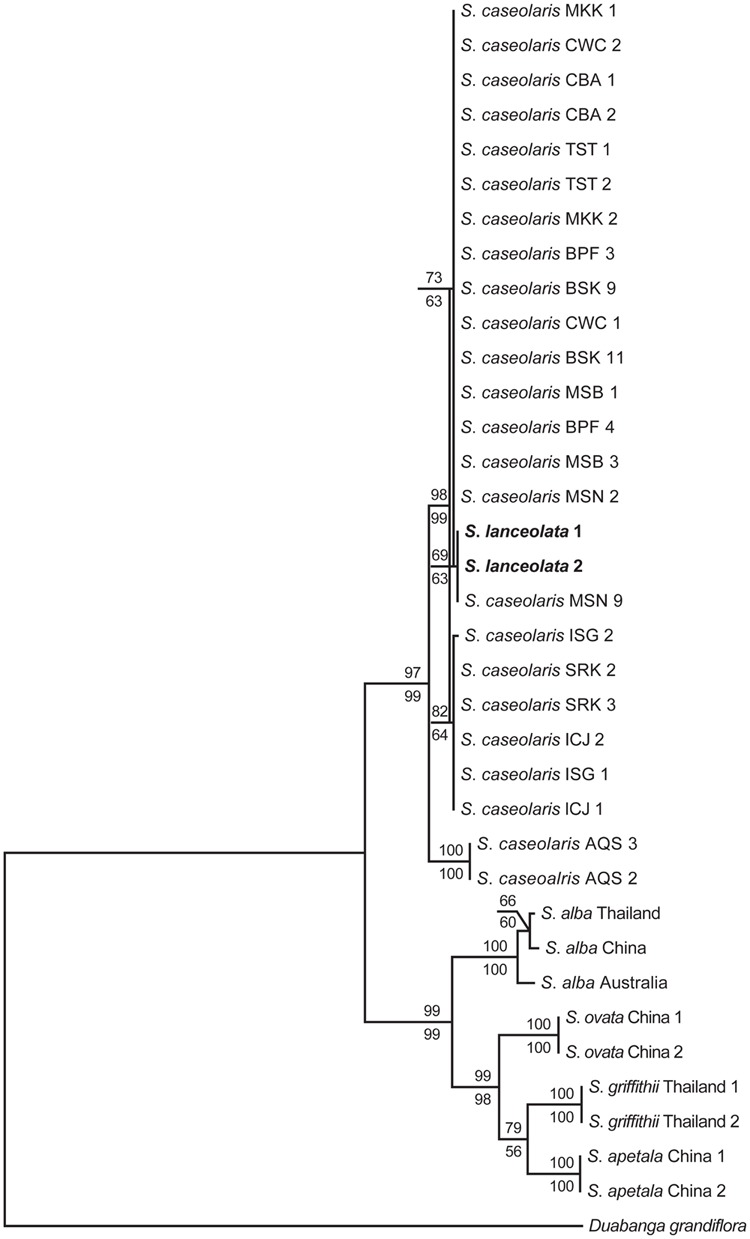
**Maximum parsimony (MP) and maximum likelihood (ML) phylogenetic trees of *Sonneratia* based on the combined sequences of nrITS and one nuclear gene *rpl9*.** *S. lanceolata* samples are shown in bold. Numbers up and down the branches are bootstrap values (>50%) of the MP and ML analysis, respectively. The abbreviations of the sampling location after species names are listed in Additional file 6: **Supplementary Table S4**.

We also estimated divergence time between *S. lanceolata* and *S. caseolaris* using the BEAST software v. 1.8.2 (Drummond et al., 2012). According to the results of Bayesian clustering and MDS analyses, the CWC population was selected to represent the SCS clade of *S. caseolaris*, while the two populations SRK and BSK were selected for the Indian Ocean clade. For each population, the major allele of each nuclear gene was selected and concatenated with alleles from other genes to represent the genetic composition of each clade. The topology of the prior tree was chosen based on the phylogenetic relationships described in **Figure 1**. The estimated divergence times of S. *ovata, S. alba*, and *S. caseolaris* were set as three calibration points under Gaussian priors with means of 7.0, 9.0, and 11.0 million years and a standard deviation of 2.0 million years, respectively, based on a separate genomic analysis of mangrove species.

### Demographic History Estimation Using the Isolation with Migration Model

To assess the level of interregional gene flow, we estimated pairwise migration rates (2NM), as well as effective population size (Ne) and divergence time (t), among populations from the SCS, Indian Ocean and the Indo-Pacific boundary under the isolation with migration (IM) model. We used data from the five nuclear genes and the IMa2 program (Hey and Nielsen, 2007; Hey, 2010). Two populations from Hainan, China (CWC and CBA) were selected to represent the SCS lineage and three populations from Sri Lanka (SRK) and Bangladesh (BSK and BPF) were chosen for the Indian Ocean lineage, according to the results of Bayesian clustering and MDS analysis (**Figure 3A**; Supplementary Figure S2). Individuals from the BSK and BPF populations were merged to increase sample size. The population from Sungaibuntu, Java, Indonesia (ISG) with putative signal of genetic admixture was included to examine whether there was substantial secondary contact at the Indo-Pacific boundary. According to our Bayesian clustering analysis, the ISG population showed a closer relationship to the Indian Ocean lineage than the SCS lineage. Thus, in our prior tree, the SCS and the Indian Ocean lineages split, and then the ISG population diverged from the Indian Ocean lineage at t2 (**Figure 5A**). The divergence time of the two SCS populations CWC and CBA was denoted as t0, while that between the populations from Sri Lanka (SRK) and Bangladesh (BSK and BPF) was represented by t1. For each gene, the longest non-recombining block was extracted from the aligned data set using the Perl script IMgc (Woerner et al., 2007) to avoid biased parameter estimation caused by recombination. The HKY substitution model was employed in this analysis. The *priori* upper bounds of demographic parameters were set to include most of their posterior distributions as determined by preliminary simulations. However, when there were no good attainable peaks, the maximum cut-off of the ancestral population size was set to 8, pairwise migration rates to 8 and divergence time to 5. Based on the results of preliminary trial runs, we ran 100 chains each with a burn-in of 1^∗^10^5^ iterations and 5^∗^10^6^ sampling steps with a geometric heating scheme (-hfg -ha0.96 -hb0.9). Three independent runs were carried out with different, random number seeds to test for convergence. The 5^∗^10^6^ well-mixed samples were thinned to obtain 100,000 draws from marginal posterior distributions of each parameter. Posterior most likely values were estimated by finding posterior modes (Nielsen and Wakeley, 2001). Due to the lack of accurately established mutation rates for either the genus *Sonneratia* or the family Lythraceae, we used the substitution rates estimated by the BEAST software for the IM analysis.

## Results

### Phylogenetic Position of *S. lanceolata*

The combined sequences of nrITS and the nuclear gene *rpl9* were 1,734 bp long after alignment, with 135 parsimoniously informative sites. The MP analysis produced one most parsimonious tree with tree length of 370, CI of 0.911 and RI of 0.933 (**Figure 1**). The ML analysis yielded the same topology as the MP method. The six *Sonneratia* species were divided into two clades with strong support, one of which grouped *S. lanceolata* and *S. caseolaris* (BS = 98% and 78% for MP and ML analysis, respectively) and the other contained the remaining four species (BS = 99% for both MP and ML analyses). Surprisingly, *S. lanceolata* was nested within the clade formed by *S. caseolaris* samples and was most closely related to the *S. caseolaris* individuals from Indo-Malesia region with high bootstrap support (BS = 97% and 96% for MP and ML analysis, respectively).

### Polymorphisms within *S. caseolaris*

**FIGURE 2:**
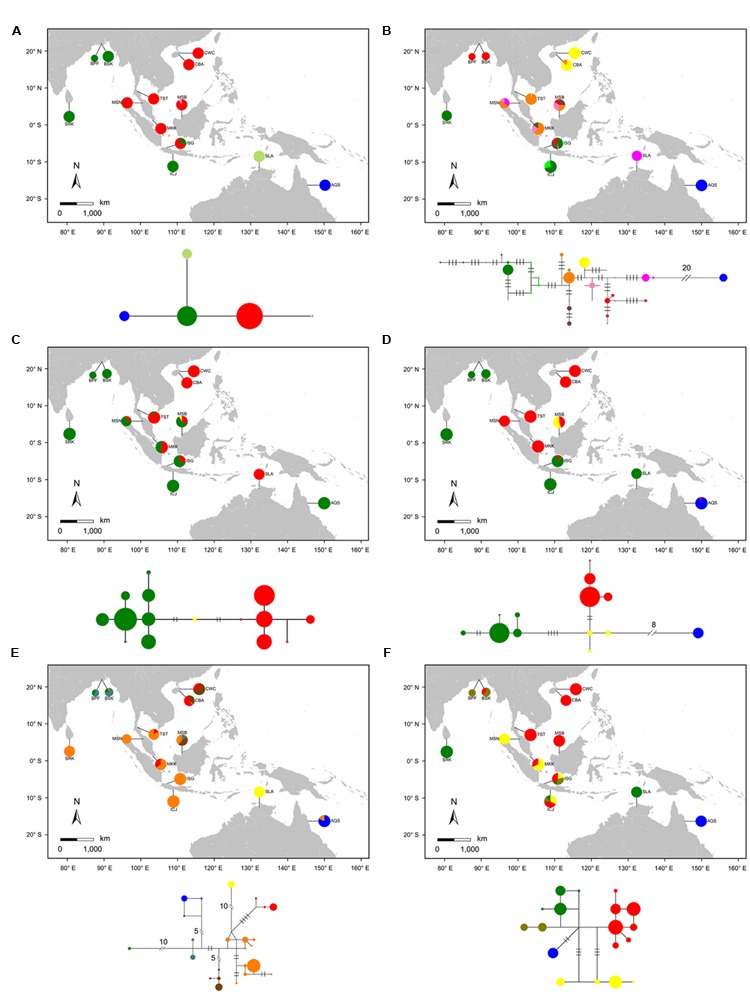
**Geographic distribution of haplotypes and Median-Joining network for the combined chloroplast fragments (**A**) and five nuclear genes (**B**: *rpl9*, **C**: *cpi*, **D**: *ppi*, **E**: *phi*, and **F**: *nhx2*) in the 12 populations of *Sonneratia caseolaris* and the single population of *S. lanceolata*.** Each haplotype was represented by a single circle whose size reflects haplotype frequency. Haplotypes with close relationships are marked with the same color. Each branch with more than one mutational step is labeled. Population abbreviations are defined in **Table 1**.

We sequenced 210 individuals from 12 populations of *S. caseolaris*. We observed two single-nucleotide polymorphisms (SNPs) and one one-bp indel in the combined chloroplast fragments. These sites generated four haplotypes across the 12 populations of *S. caseolaris* (**Figure 2A**). Only two populations (MSB and ISG) of *S. caseolaris* were polymorphic with extremely low levels of nucleotide diversity (𝜃_π_) and polymorphisms (𝜃_w_, **Supplementary Table S3**).

The aligned sequence length of the five nuclear genes ranged from 620 to 1,170 bp, containing 12 to 49 SNPs in the 12 populations. At the species level, *S. caseolaris* harbored relatively high levels of genetic diversity, with 𝜃_π_ ranging from 3.660 to 8.850 per kb, and 𝜃_w_ from 2.820 to 5.560 per kb (Supplementary Figure S1; **Supplementary Table S3**). At the population level, populations of *S. caseolaris* from its range margins (CWC, SRK, BPF, BSK, and AQS) exhibited lower levels of polymorphism than those from its distribution center (MSB, MKK, MSN, ISG, and ICJ, Supplementary Figure S1). MSB and ISG populations had the highest levels of nucleotide diversity (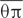 = 3.556 and 3.558 per kb, respectively), while the population from Sri Lanka (SRK) had the lowest diversity (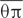 = 0.054 per kb).

### Population Structure within *S. caseolaris* Across the IWP Region

Haplotype network analyses at both the chloroplast fragments and the five nuclear genes showed strong population structure that tracked with geographical distribution of *S. caseolaris* (**Figure 2**). Four chlorotypes in the 12 populations fell into three major clusters corresponding to three geographic regions. The first cluster comprises populations around the SCS (CWC, CBA, TST, MSB, MKK, and MSN), the second is restricted to the Indian Ocean (SRK, BPF, BSK, and ICJ), and the third is found exclusively in Queensland, Australia (AQS, **Figure 2A**). Similar to the chloroplast fragments, haplotype networks of four of the five nuclear genes (*rpl9, ppi, phi*, and *nhx2*) showed three distinct groups restricted to the SCS, the Indian Ocean and eastern Australia (**Figures 2B,D,E,F**). Polymorphisms in the *cpi* gene revealed only two groups, with one predominant in the SCS and the other combining the Indian Ocean and Australian populations (**Figure 2C**). The haplotype networks, in addition to a MDS analysis of the genetic distance matrix, showed that the population from eastern Australia was most highly diverged from the SCS and the Indian Ocean (**Figure 2**; Supplementary Figure S2). This pattern is consistent with early separation of the Indo-Malesia from Australasia.

**FIGURE 3:**
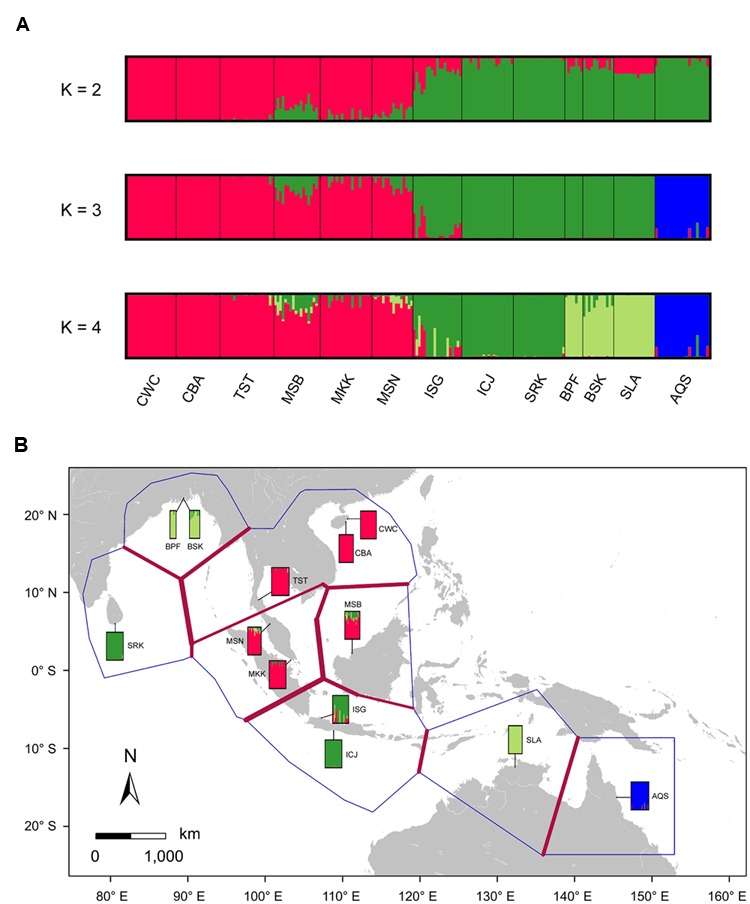
**Likely genetic clusters and geographical barriers existing among the 12 populations of *S. caseolaris* and the single population of *S. lanceolata*. (A)** Bayesian clustering analysis of the five nuclear genes implemented in STRUCTURE. To illustrate the hierarchical population structure across the IWP region, we show clustering with the best-fitting model of *K* = 2, as well as *K* = 3 and *K* = 4. **(B)** Putative geographical barriers identifed within the IWP regions. The bars show the output of Bayesian clustering analysis with *K* = 4. Population abbreviations are defined in **Table 1**.

Bayesian clustering analysis using STRUCTURE suggests that the statistically optimal number of populations is *K* = 2 (Supplementary Figure S3). However, the results for *K* = 3 and 4 were also biologically meaningful and were included in this study. At *K* = 3 Bayesian clustering analysis generated population assignments consistent with haplotype network and MDS analysis, splitting samples from the SCS, the Indian Ocean and eastern Australia into their own groups (**Figure 3A**; Supplementary Figure S2). Using the Barrier software, strongest barriers, supported by four of the five genes, were identified between the populations from the SCS and the Indian Ocean, corresponding to the high genetic divergence of the SCS and the Indian Ocean lineages (**Figure 3B**). Within the Indian Ocean group, a relatively weak barrier marked a subdivision between Sri Lanka and Bangladesh, which was in agreement with the Bayesian clustering result with *K* set to 4. These results indicated that geographic features in the IWP region might have profoundly shaped population structure within *S. caseolaris*.

**FIGURE 4:**
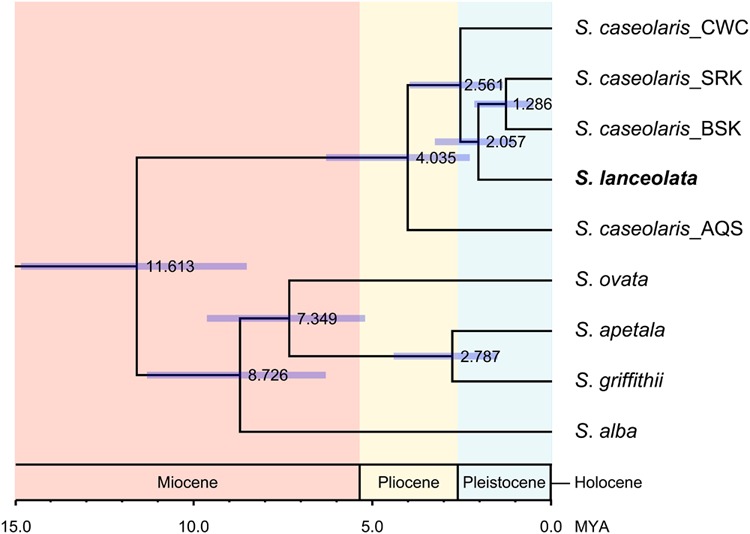
**Divergence time of the six *Sonneratia* species, as well as the different lineages of *S. caseolaris*.** The scale bar is 1.0 million years (MYA). The value and purple bar at each node indicate the estimated divergence time (MYA) with its 95% highest probability density interval (HPDI).

Within *S. caseolaris*, the divergence time between the Indo-Malesia and Australasia populations was estimated to be 4.035 million years ago (MYA; 95% of the highest probability density interval (HPDI): 2.298–6.318 MYA, **Figure 4**). Within the Indo-Malesia region, the split between the SCS and the Indian Ocean lineages occurred at 2.561 MYA (95% HPDI: 1.375–3.981 MYA), while the Sri Lanka population diverged from Bangladesh (BSK and BPF) 1.286 MYA (95% HPDI: 0.522–2.174 MYA).

### Genetic Admixture at the Indo-Pacific Boundary

We observed extensive admixture between the SCS and the Indian Ocean groups, as evidenced by the analyses of chloroplast fragments as well as nuclear genes, in the Sangaibuntu population (ISG), located near the Indo-Pacific boundary. Similar patterns were also observed in three other populations that are close to the Malacca Strait (MSB, MKK, and MSN). Bayesian clustering likewise showed signals of genetic admixture between the SCS and the Indian Ocean groups in the ISG, MSB, and MSN populations (**Figure 3A**). When *K* was set to 2, approximately 74.6% of the nucleotide variations present in the ISG population came from the Indian Ocean lineage, while 25.4% were from the SCS lineage. In the two Malaysian populations, the proportion of SNPs that belong to the SCS lineages was slightly higher (78.1 and 87.3% in MSB and MSN, respectively).

**FIGURE 5:**
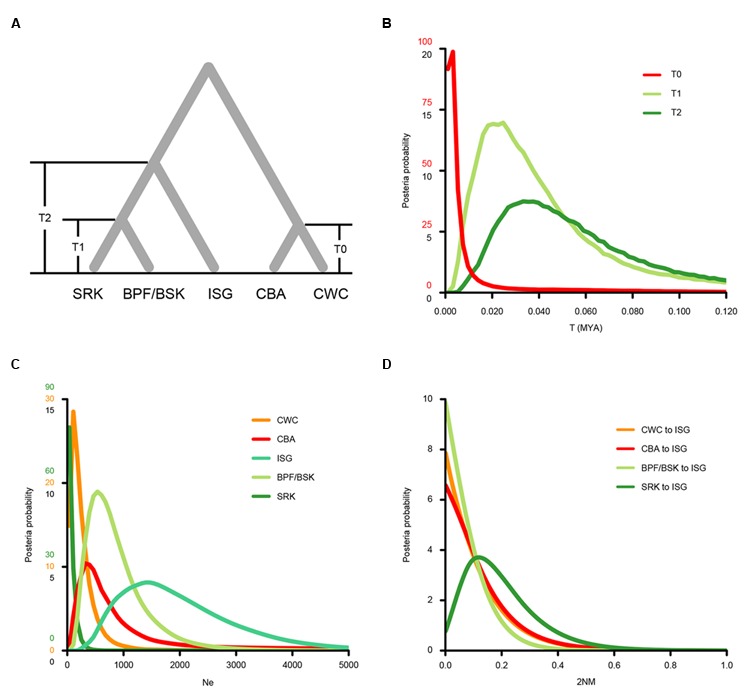
**Probability density plots of the demographic parameters estimated from the isolation with migration model for populations from the SCS (CWC and CBA), the Indian Ocean (SRK, BSK and BPF) and the Indo-Pacific boundary region (ISG). (A)** A schematic of the isolation with migration model. Population abbreviations are defined in **Table 1**. **(B)** Probability density of the divergence time between CWC and CBA (T0), between the Sri Lanka population (SRK) and two Bangladesh populations (BSK and BPF, T1) and the formation time of the ISG population (T2). **(C)** Probability density of the effectively population sizes (Ne) of the five populations. **(D)** Probability density estimation of the migration rate from the CWC, CBA, BSK and BPF and SRK to ISG populations.

We then used the isolation with migration (IM) model to estimate demographic parameters of *S. caseolaris* populations from the SCS (CWC and CBA), the Indian Ocean (BSK, BPF and SRK) and the Indo-Pacific boundary (ISG) (**Figure 5**; **Table 2**). The effective sample size (ESS) was larger than 70,000, indicating a well-mixed chain. As a result of running the BEAST program on our data, we obtained an estimate of the mutation rate at 1.673 ^∗^ 10^-9^ substitutions per site per year (s/s/y; 95% HPDI: 1.219 ^∗^ 10^-9^–2.195 ^∗^ 10^-9^ s/s/y). Under this assumed mutation rate, effective size (Ne) of the two SCS populations (CWC and CBA) was estimated to be 107 (95% HPDI: 0–1,253) and 322 (95% HPDI: 0–64,092), respectively; Ne of the populations from the Indian Ocean (BSK/BPF and SRK) was 537 (95% HPDI: 179–2,111) and 36 (95% HPDI: 0–680), respectively (**Figure 5C**). Ne of the ISG population was estimated at 1,396 (95% HPDI: 394–4,187), much greater than that in other populations. The migration rate from the Indian Ocean population SRK to the population ISG was estimated to 0.119 (95% HPDI: 0.000–0.452), which was significantly greater than zero (LLR = 3.224, *p* < 0.05, **Figure 5D**).

**Table 2:** MLEs and the 95% highest probability density interval (HPDI) of all demographic parameters estimated using the isolation with migration model (IMa2).

Mutation rate (^∗^ 10^-9^ s/s/y)	T0 (MYA)	T1 (MYA)	T2 (MYA)	Ne_CWC_	Ne_CBA_	Ne_ISG_	Ne_BSK/BPF_	Ne_SRK_	2NM_CWC→ISG_	2NM_CBA→ISG_	2NM_BPF/BSK→ISG_	2NM_SRK→ISG_
1.219	0.004	0.034	0.046	147	442	1,915	737	49
	(0.000–0.090)	(0.004–0.299)	(0.007–2.108)	(0–1,719)	(0–87,962)	(540–5,747)	(246–2,897)	(0–933)
1.673	0.003	0.025	0.033	107	322	1,396	537	36	0.001	0.001	0.001	0.119
	(0.000–0.065)	(0.003–0.218)	(0.005–1.536)	(0–1,253)	(0–64,092)	(394–4,187)	(179–2,111)	(0–680)	(0.000–0.347)	(0.000–0.354)	(0.000–0.233)	(0–0.452)
2.195	0.002	0.019	0.025	82	245	1,064	409	27
	(0.000–0.050)	(0.002–0.166)	(0.004–1.171)	(0–955)	(0–48,850)	(300–3,191)	(136–1,609)	(0–518)
LLR									-0.000	-0.000	-0.000	3.224^∗^

*For each parameter, 95% HPDI is shown in parentheses. T0 is the divergence time between the CWC and CBA populations, while T1 is the divergence time between the populations from Sri Lanka (SRK) and Bangladesh (BSK and BPF). T2 represents the formation time of the ISG population. Mutation rate is 1.673^∗^10^-9^ substitutions per site per year (s/s/y; 95% HPDI: 1.219 ^∗^ 10^-9^–2.195 ^∗^ 10^-9^ s/s/y). “^∗^” denotes *p* < 0.05 for the migration rate likelihood ratio test.*

### Genetic Divergence between *S. caseolaris* and *S. lanceolata*

In contrast to *S. caseolaris*, genetic diversity of the *S. lanceolata* population (SLA) was extremely low in both chloroplast fragments and the five nuclear genes, with θ_π_ ranging from 0.000 to 0.110 and θ_w_ from 0.000 to 0.350 per kb (**Supplementary Table S3**).

The position of *S. lanceolata* within the clade that comprises the *S. caseolaris* samples suggested by our phylogenetic analysis was verified by the haplotype network reconstruction and Bayesian clustering. No polymorphism was detected in the chloroplast fragments sequenced in *S. lanceolata*, and this species differs from the Indian Ocean populations of *S. caseolaris* by only one mutation (**Figure 2A**). At the two of the five nuclear genes (*ppi* and *nhx2*), *S. lanceolata* is most closely related to the Indian Ocean populations of *S. caseolaris* (**Figures 2D,F**). At the gene *phi*, the haplotype of *S. lanceolata* forms its own clade that differs from the Indian Ocean *S. caseolaris* samples by 11 mutations (**Figure 2E**). In contrast, the remaining two genes, *rpl9* and *cpi*, contained haplotypes that grouped with the Malaysia population (MSN) of *S. caseolaris* (**Figures 2B,C**).

For the optimal clustering (*K* = 2, Supplementary Figure S3) of the Bayesian clustering, *S. lanceolata* was assigned to the same cluster as the Indian Ocean and Australian populations of *S. caseolaris*, distinct from the SCS group of that species (**Figure 3A**). When *K* = 3, there was a division between the *S. caseolaris* populations from the Indian Ocean and Australia of *S. caseolaris* with the *S. lanceolata* population falling into the Indian Ocean population cluster. Increasing *K* to 4 results in *S. lanceolata* samples forming a new cluster with the two Bangladesh populations of *S. caseolaris*. These results suggest that *S. lanceolata* is closely related to the Indian Ocean lineage of *S. caseolaris*. Moreover, based on the pairwise *K*_XY_ values (Supplementary Figure S4; **Supplementary Table S4**), two putative barriers to gene flow between *S. caseolaris* and *S. lanceolata* were identified. One is located between northern Australia and Java Island, and the other is between the Arafura Sea and the Coral Sea. These two geographic barriers surround the current distribution region of *S. lanceolata* (**Figure 3B**). This suggests that the isolation of the Arafura Sea from the surrounding oceanic regions might have triggered the *S. lanceolata* speciation event. Divergence time estimates indicates that *S. lanceolata* diverged from the Indian Ocean lineage of *S. caseolaris* at approximately 2.057 MYA with 95% HPDI extending from 1.053 to 3.275 MYA (**Figure 4**).

## Discussion

### Geographic Isolation Drives Strong Genetic Differentiation among *S. caseolaris* Populations

Strong genetic differentiation among the *S. caseolaris* populations was associated with geographic barriers. We identified three distinct genetic lineages located in the SCS, Indian Ocean and Australia (**Figures 2** and **3A**; Supplementary Figure S2). We dated the split between the Indo-Malesia and the Australasia regions to 4.035 MYA (95% HPDI: 2.298–6.318 MYA, **Figure 4**), prior to the Pleistocene. This result suggests a critical role for pre-Pleistocene events in genetic divergence of the two main lineages of *S. caseolaris*.

We further estimated that the SCS lineage diverged from the Indian Ocean lineage 2.561 MYA (95% HPDI: 1.375–3.981 MYA), in the early Pleistocene. Perhaps sea level changes during this epoch drove the genetic differentiation between populations from these geographic areas. Dispersal of seeds via sea currents plays an important role in determining the extant distribution and genetic composition of mangrove species within and among different oceanic regions (Duke, 1995; Dodd and Rafii, 2002; Triest, 2008). During glacial periods, global sea levels dropped and exposed the bulk of the shallow seabed in the SCS, forming temporary land bridges connecting mainland Asia with the three Sunda Islands (Sumatra, Borneo and Java; Voris, 2000; Sathiamurthy and Voris, 2006). This landmass likely acted as a strong physical barrier halting sea-drifted gene flow between the SCS and the Indian Ocean populations of *S. caseolaris* during Pleistocene, consistent with the differentiation mechanism suggested for other mangrove species (Ge and Sun, 2001; Liao et al., 2007; Huang et al., 2008; Urashi et al., 2013; Wee et al., 2015) and numerous marine organisms (Barber et al., 2000; Nelson et al., 2000; Crandall et al., 2008; DeBoer et al., 2008; Froukh and Kochzius, 2008).

It is usually difficult to distinguish genetic admixture from incomplete lineage sorting, thus reducing the resolution of signals of gene flow (Ng et al., 2015). In this study, we provide novel evidence for genetic admixture of the SCS and Indian Ocean lineages at the Indo-Pacific boundary in *S. caseolaris*. Analyses of haplotype frequencies at the chloroplast fragments and four of the five nuclear genes showed that the ISG population harbors haplotypes from both the SCS and the Indian Ocean groups (**Figures 2B–D,F**). Haplotypes of nuclear genes fell into two highly divergent clusters separated from each other by 5–14 mutational steps and there were no intermediate haplotypes. This pattern is highly unlikely to have arisen within ISG in the face of ancestral polymorphism, strongly suggesting that previously isolated lineages came into contact and mixed in this population. This idea is consistent with our simulation under the isolation with migration (IM) model that identified substantial gene flow from the Sri Lanka population (SRK) into ISG (**Figure 5D**).

Although the basin of the Java Sea was exposed during glaciations, especially the Last Glacial Maximum (LGM), subsequent interglacial sea-level rises restored the sea corridors connecting the SCS and the Indian Ocean (Wyrtki, 1961). It is thus easily to imagine that seeds from both the SCS and the Indian Ocean could rapidly re-colonize the seashore of the Java Sea via drift on sea currents, resulting in secondary contact of the two previously isolated lineages in this area. Similar genetic admixture of SCS and Indian Ocean groups was observed in two mangrove species *Rhizophora apiculata* (Yahya et al., 2014) and *Lumnitzera racemosa* (Li et al., 2016), as well as the false clown anemonefish (*Amphiprion ocellaris*, Timm and Kochzius, 2008) and reef communities (Hoeksema, 2007).

### Recent Origin of *S. lanceolata* Triggered by Glacial Isolation

*S. lanceolata* was first described as a new species by Blume (1851) and is highly similar to *S. caseolaris* in both morphology and ecology, suggesting a close relationship between these two species (Duke and Jackes, 1987; Duke et al., 1998). Coincidently, our phylogenetic analysis using the combined sequences of nrITS and the nuclear gene *rpl9* indicates that *S. lanceolata* is nested within the *S. caseolaris* clade (**Figure 1**). The main differentiating character between these species is stamen color, which is white in *S. lanceolata* (Duke and Jackes, 1987; Duke, 1994, 2006). However, the stamen colors of *S. caseolaris* vary in different locations. For example, the stamens of *S. caseolaris* are purplish red in Australia and Bangladesh, while they are white in the upper part and red in the basal part in China and Thailand (S. Shi, personal observation). Bayesian clustering analysis and K_XY_ statistics showed that *S. lanceolata* individuals (collected in Australia) were genetically closer to the Indian Ocean group than either the SCS or Australia groups of *S. caseolaris* (**Figure 3A**; Supplementary Figure S4). The split between these two species was estimated to occur 2.057 MYA (95% HPDI: 1.053–3.275 MYA, **Figure 4**), during early Pleistocene.

Our results of genetic similarity and divergence time are consistent with an important role of geographic isolation driven by Pleistocene glaciations as a trigger of lineage break between *S. lanceolata* and *S. caseolaris*. At the boundary between Indo-Malesia and Australasia, over 25,000 volcanic ocean islands and the shallow seas surrounding them are particularly sensitive to sea level fluctuations (Briggs, 1987, 2005; Bellwood et al., 2004; Hoeksema, 2007). During Pleistocene glaciations, sea levels dropped as much as 116 m (Miller et al., 2005; Sathiamurthy and Voris, 2006), connecting the islands and forming a strong physical barrier that likely prevented genetic exchange between the two regions (Triest, 2008; Esselstyn et al., 2009; Lohman et al., 2011). Likewise, the Torres Strait is a shallow watercourse connecting the Arafura Sea and the Coral Sea (only 7–15 m deep; Harris, 1988) that spent approximately 70% of the time since the Pleistocene closed to sea traffic (Miller et al., 2005). These two barriers to gene flow likely enabled the split between *S. lanceolata* and *S. caseolaris*.

Similar genetic differentiation and speciation might have also occurred in other mangrove genera. *Avicennia integra* is a narrowly distributed species confined to the Northern Territory, Australia (Duke, 2006). In contrast, its sister species *A. officinalis* has a much wider range extending from South India through Malaysia and Indonesia to New Guinea and eastern Australia (Tomlinson, 1986). The divergence time between these two species was estimated at 2.5 MYA, corresponding to the onset of Pleistocene (Li, 2015). Another case was observed between two sibling species of *Ceriops, C. tagal* and *C. australis*. Morphologically, *C. australis* differs from *C. tagal* by the shorter terete and smooth hypocotyls and longer flowers, calyx lobes and petals (White, 1926; Ballment et al., 1988; Sheue et al., 2009). Geographically, *C. australis* is restricted to Australia, along the costal lines of North Territory and Queensland, and a part of New Guinea and Indonesia, while *C. tagal* widely occurs from southern India through Southeast Asia to Australia (Duke, 2006). However, *C. australis* is found more often than *C. tagal* in Australia (Sheue et al., 2009). These findings suggested that Pleistocene glacial vicariance may have been a general force contributing to recent inter-species divergence among numerous mangrove genera.

## Author Contributions

SS and RZ designed the study. YY, FP, and CZ collected materials and performed experiments. YY, FP, JL, and SY analyzed and interpreted the data. YY, ND, FP, RZ, and SS wrote the manuscript. All authors read and approved the final manuscript.

## Conflict of Interest Statement

The authors declare that the research was conducted in the absence of any commercial or financial relationships that could be construed as a potential conflict of interest.

The reviewer YS declared a shared affiliation, though no other collaboration, with several of the authors YY, FP, JL, SY, RZ, SS to the handling Editor, who ensured that the process nevertheless met the standards of a fair and objective review.

## Footnotes

1 https://www.r-project.org/

## Supplementary Material

TABLE S1 **Gene IDs, gene descriptions, primer sequences and GenBank accession numbers of haplotypes for the two chloroplast fragments and five nuclear genes used in this study**.

TABLE S2 **GenBank accession numbers of the sequences of the nuclear ribosomal internal transcribed spacer (nrITS) and the nuclear gene *rpl9* for the six *Sonneratia* species and the outgroup *D. grandiflora* used in this study**.

TABLE S3 **Statistics of the combined chloroplast fragments and the five nuclear genes for the populations of *S. caseolaris* and *S. lanceolata*.** Population IDs are defined in **Table 1**. S, the number of segregating sites; h, the number of haplotypes; Hd, haplotype diversity; θπ, nucleotide diversity; θw, DNA polymorphism.

TABLE S4 **Means and standard variations (SD) of pairwise *K*_XY_ values of the five nuclear genes among the 13 populations of *S. caseolaris* and *S. lanceolata*.** Means were listed in lower left matrix, while SD were listed in the upper right matrix. Population abbreviations are defined in **Table 1**.
