# Supplementary material for: Ancient Geographical Barriers Drive Differentiation among Sonneratia caseolaris Populations and Recent Divergence from S. lanceolata
Source: Front Plant Sci. 2016 Oct 26;7:1618. doi: 10.3389/fpls.2016.01618 (PMC5080369; doi:10.3389/fpls.2016.01618)
Supplement: Supplementary file 5 [file Image_1.PDF]

Supplementary Materials

Ancient geographical barriers drive differentiation among *Sonneratia caseolaris* populations and recent divergence from *S. lanceolata*

Yuchen Yang[1], Norman C. Duke[2], Fangfang Peng[1], Jianfang Li[1], Shuhuan Yang[1], Cairong Zhong[3], Renchao Zhou[1]*, Suhua Shi[1]*

* Corresponding author
Renchao Zhou
zhrench@mail.sysu.edu.cn
Suhua Shi
lssssh@mail.sysu.edu.cn

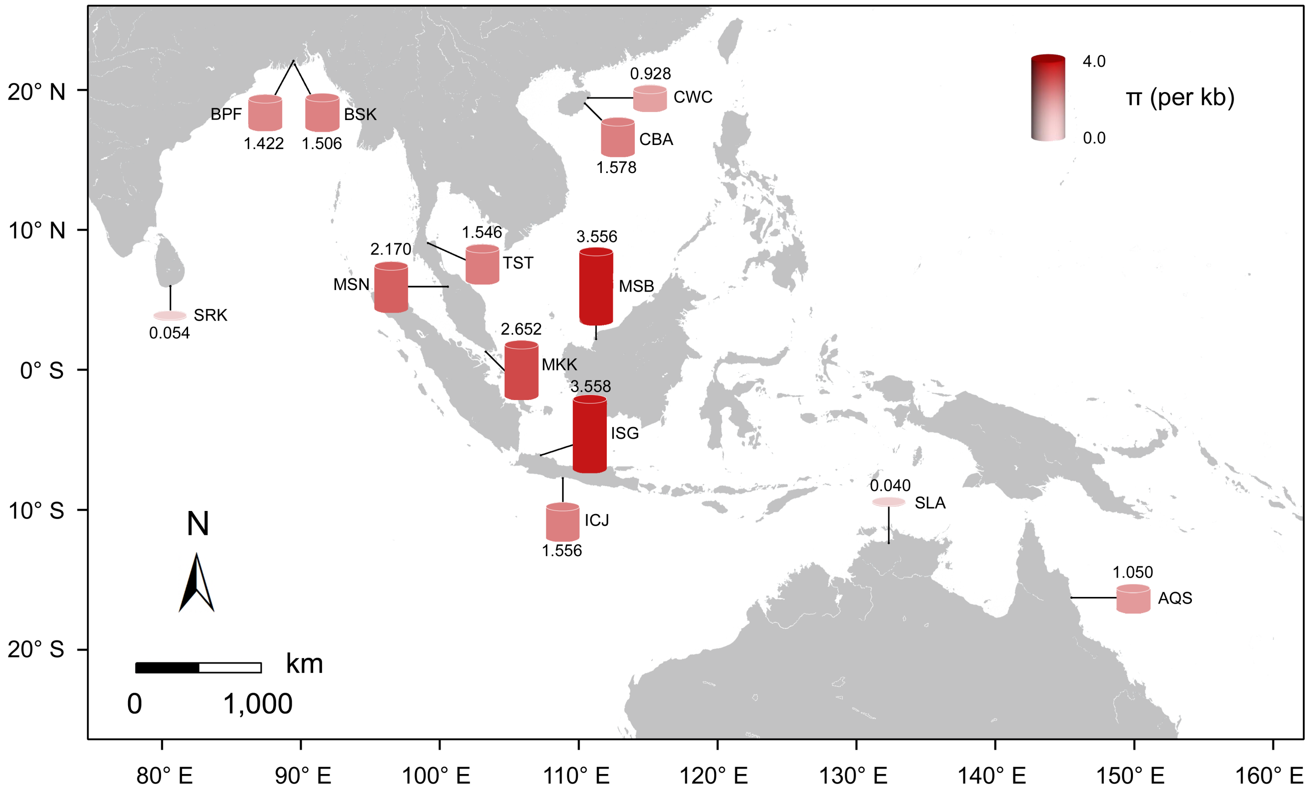

Figure S1. A heatmap of nucleotide diversity (θπ) of the 12 *Sonneratia caseolaris* populations and the single *S. lanceolata* population. The color depth and the height of the cylinder are proportional to the level of of θπ. Population abbreviations are defined in Table 1.

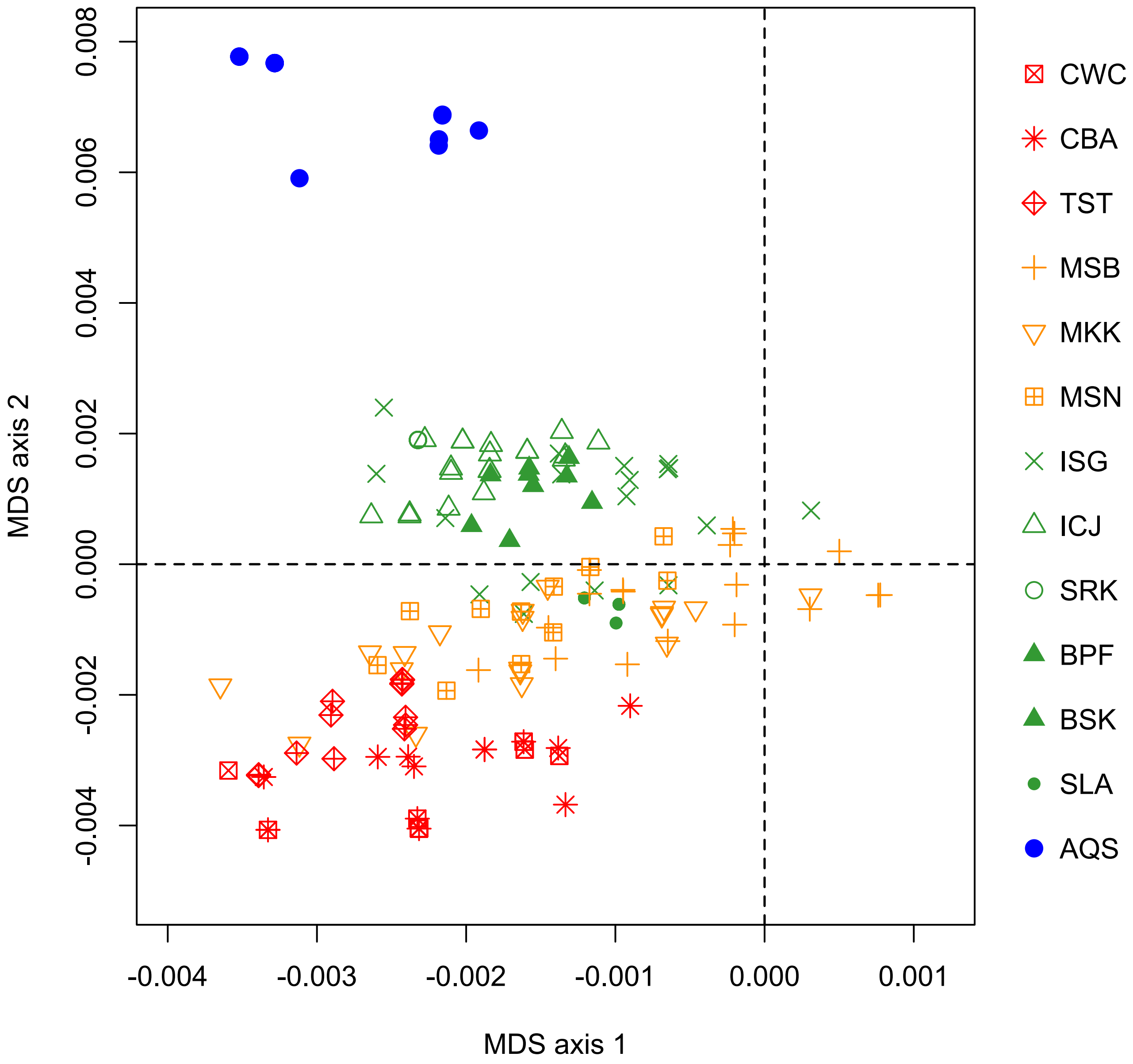

Figure S2. Multidimensional scaling (MDS) analysis of the individuals from the 12 populations of *Sonneratia caseolaris* and the single population of *S. lanceolata*. Population abbreviations are defined in Table 1.

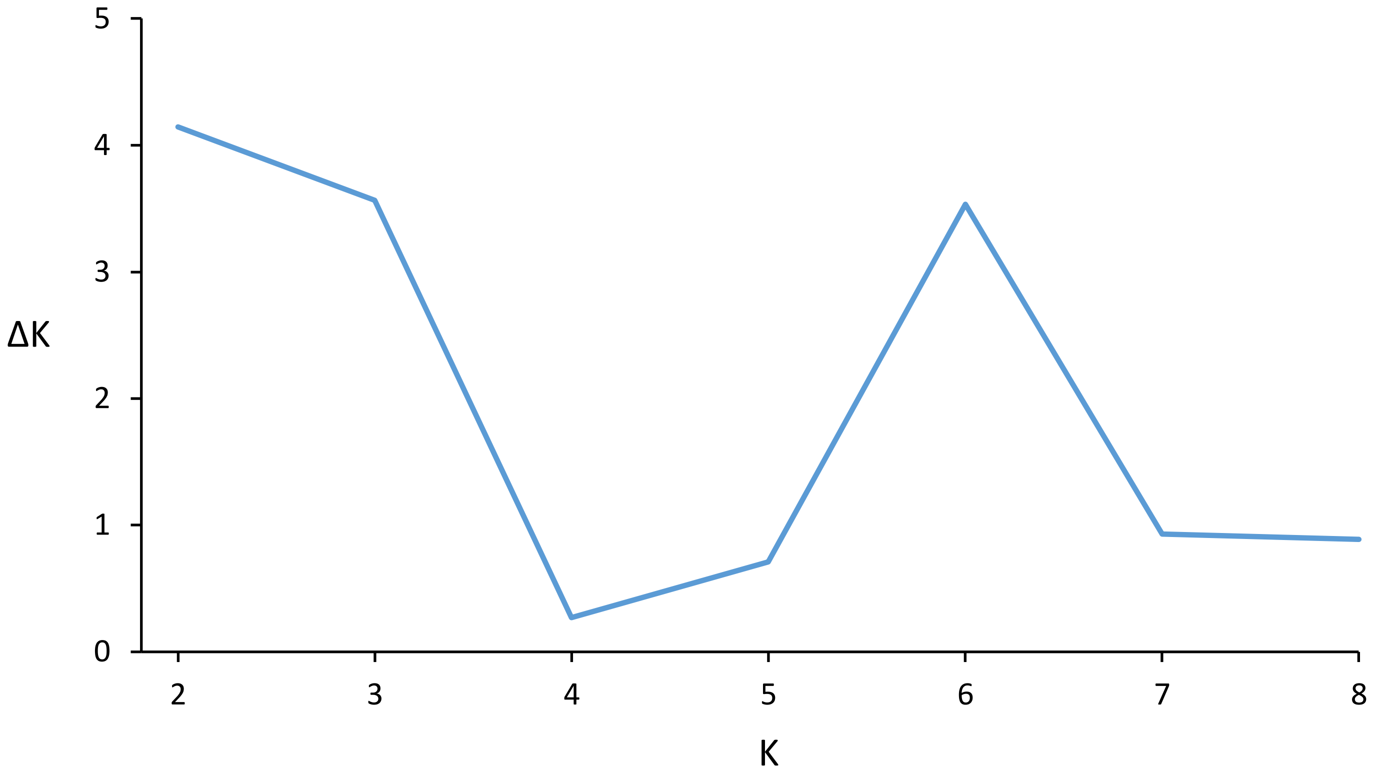

Figure S3. Diagram for inferring the optimal K obtained by STRUCTURE across the 12 *Sonneratia caseolaris* populations and one *S. lanceolata* population using ΔK statistic of Evanno et al. (2005).

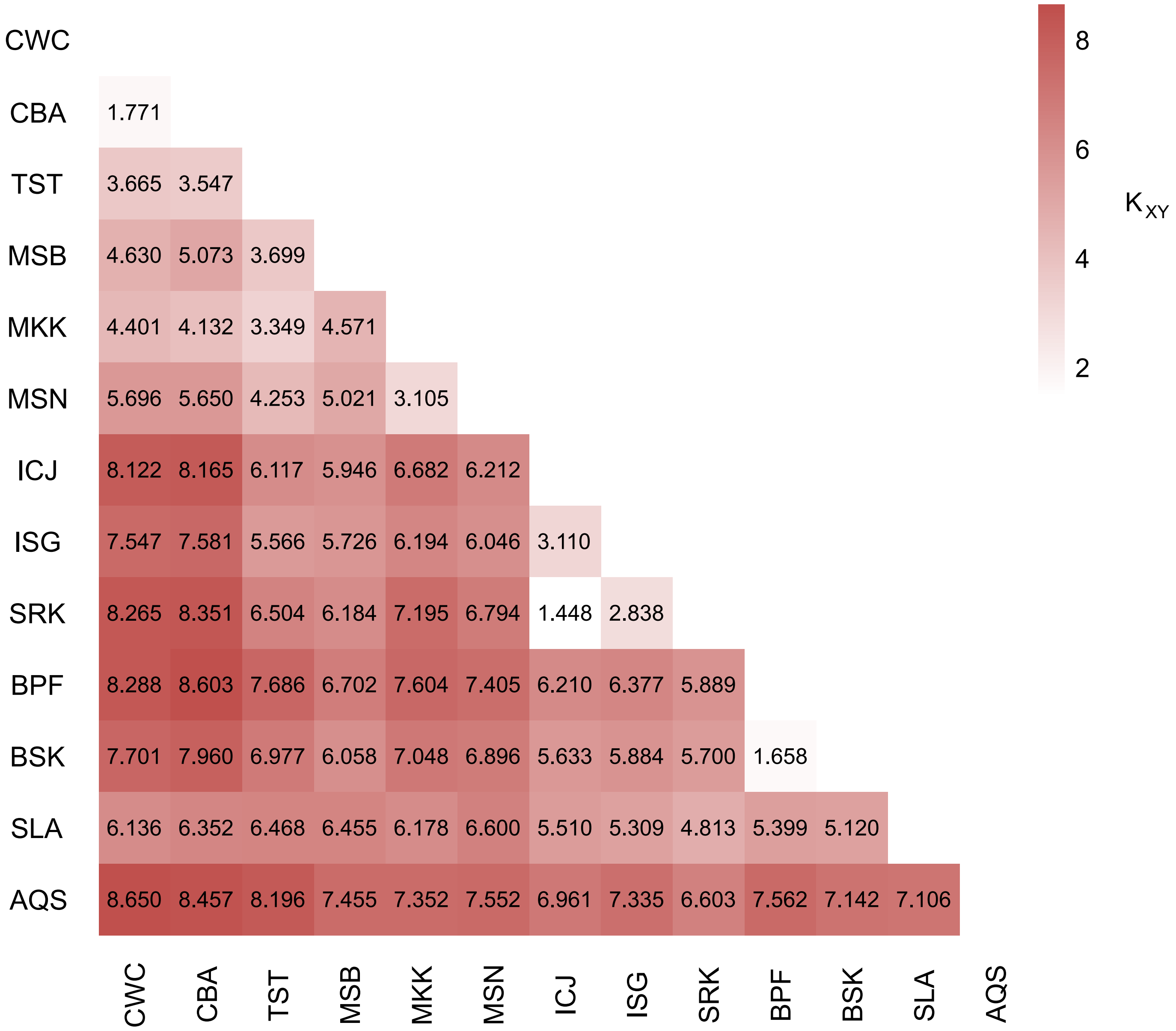

Figure S4. A heatmap of the means of pairwise Kxy values of the five nuclear genes among 13 populations of *Sonneratia caseoalris* and *S. lanceolata*. The colour depth was proportional to the level of genetic differentiation. Population abbreviations are defined in Table 1.
